# Point-of-Care Digital Cytology With Artificial Intelligence for Cervical Cancer Screening in a Resource-Limited Setting

**DOI:** 10.1001/jamanetworkopen.2021.1740

**Published:** 2021-03-17

**Authors:** Oscar Holmström, Nina Linder, Harrison Kaingu, Ngali Mbuuko, Jumaa Mbete, Felix Kinyua, Sara Törnquist, Martin Muinde, Leena Krogerus, Mikael Lundin, Vinod Diwan, Johan Lundin

**Affiliations:** 1 Institute for Molecular Medicine Finland, University of Helsinki, Helsinki, Finland; 2 Department of Women's and Children’s Health, International Maternal and Child Health, Uppsala University, Uppsala, Sweden; 3 Kinondo Kwetu Health Services Clinic, Kinondo, Kenya; 4 Department of Global Public Health, Karolinska Institutet, Stockholm, Sweden; 5 Helsinki University Central Hospital Laboratory (HUSLAB), HUS Diagnostic Center, Helsinki and Uusimaa Hospital District, Helsinki, Finland

## Abstract

**Importance:**

Cervical cancer is highly preventable but remains a common and deadly cancer in areas without screening programs. The creation of a diagnostic system to digitize Papanicolaou test samples and analyze them using a cloud-based deep learning system (DLS) may provide needed cervical cancer screening to resource-limited areas.

**Objective:**

To determine whether artificial intelligence–supported digital microscopy diagnostics can be implemented in a resource-limited setting and used for analysis of Papanicolaou tests.

**Design, Setting, and Participants:**

In this diagnostic study, cervical smears from 740 HIV-positive women aged between 18 and 64 years were collected between September 1, 2018, and September 30, 2019. The smears were digitized with a portable slide scanner, uploaded to a cloud server using mobile networks, and used to train and validate a DLS for the detection of atypical cervical cells. This single-center study was conducted at a local health care center in rural Kenya.

**Exposures:**

Detection of squamous cell atypia in the digital samples by analysis with the DLS.

**Main Outcomes and Measures:**

The accuracy of the DLS in the detection of low- and high-grade squamous intraepithelial lesions in Papanicolaou test whole-slide images.

**Results:**

Papanicolaou test results from 740 HIV-positive women (mean [SD] age, 41.8 [10.3] years) were collected. The DLS was trained using 350 whole-slide images and validated on 361 whole-slide images (average size, 100 387 × 47 560 pixels). For detection of cervical cellular atypia, sensitivities were 95.7% (95% CI, 85.5%-99.5%) and 100% (95% CI, 82.4%-100%), and specificities were 84.7% (95% CI, 80.2%-88.5%) and 78.4% (95% CI, 73.6%-82.4%), compared with the pathologist assessment of digital and physical slides, respectively. Areas under the receiver operating characteristic curve were 0.94 and 0.96, respectively. Negative predictive values were high (99%-100%), and accuracy was high, particularly for the detection of high-grade lesions. Interrater agreement was substantial compared with the pathologist assessment of digital slides (κ = 0.72) and fair compared with the assessment of glass slides (κ = 0.36). No samples that were classified as high grade by manual sample analysis had false-negative assessments by the DLS.

**Conclusions and Relevance:**

In this study, digital microscopy with artificial intelligence was implemented at a rural clinic and used to detect atypical cervical smears with a high sensitivity compared with visual sample analysis.

## Key Points

**Question:**

Can point-of-care digital microscopy with artificial intelligence–based sample assessment be implemented at a clinic in a resource-limited setting where access to pathologists is limited and used to analyze Papanicolaou test results?

**Findings:**

In this proof-of-concept diagnostic study, Papanicolaou test results from 740 women were collected, digitized at a rural clinic in Kenya, and analyzed with a deep learning algorithm to detect atypical samples. The sensitivity for detection of atypia was high (96%-100%), with higher specificity for high-grade lesions (93%-99%) than for low-grade lesions (82%-86%), and no slides manually classified as high grade were incorrectly classified as negative.

**Meaning:**

The results of this study suggest that advanced digital microscopy diagnostics, supported by artificial intelligence, are feasible to use in rural, resource-limited settings for detection of abnormal cells in Papanicolaou tests.

## Teaser

This diagnostic study uses data from a single health center in Kenya to investigate the use of digital microscopy and artificial intelligence in a resource-limited area to detect abnormal cells in Papanicolaou tests.

## Introduction

Inadequate access to microscopy diagnostics is a problem in limited-resource areas and impairs the diagnosis of common and treatable conditions.^1^ Although significant advances have been made in digital microscopy diagnostics at the point of care (POC), their clinical implementation has been slow.^2^ Here, we propose a digital diagnostics system in which microscopy slides are digitized at the POC and uploaded using local data networks for analysis with an artificial intelligence model based on deep learning. Cervical cancer remains a common and deadly cancer in areas without screening programs.^3^ During the next decade, the disease incidence is expected to increase, and the yearly mortality is expected to double, with the largest burden of disease occurring in sub-Saharan Africa.^4^ Ultimately, vaccinations against human papillomavirus (HPV)^5^ have the potential to significantly reduce the disease incidence, but given that the full benefits of even the most efficient vaccination programs will take decades to be fully realized, millions of women remain at risk.^6^ Therefore, screening tests remain essential,^7^ and innovative POC diagnostic solutions are needed.^8^ Conventional cytology screening (Papanicolaou test analysis) can drastically reduce the incidence and mortality of cervical cancer, but the manual analysis of samples is labor intensive,^9^ is prone to variations in sensitivity and reproducibility, and requires medical experts to analyze the samples^10,11^; this makes the process difficult to implement in resource-limited settings.^12^ Human papillomavirus infections, which are the causative agent for cervical cancer, can be detected using polymerase chain reaction assays with high sensitivity and reproducibility. However, because most HPV infections are transient, the specificity for precancerous lesions is low.^13,14^ In high-resource areas, both molecular- and cytology-based screening methods are commonly used and are often combined (ie, cotesting) to improve the diagnostic accuracy.^15,16^ Digital methods have been proposed to facilitate the visual analysis of Papanicolaou tests, but the development of fully automated systems has been challenging.^17,18^ Although semiautomated systems for Papanicolaou test screening have been developed,^19^ they are limited by the need for bulky, expensive laboratory equipment^20,21,22^ and are not suitable for use at the POC or in resource-limited settings.

Recently, deep learning–based algorithms have been used for a large number of medical image-analysis applications, with levels of performance even surpassing human experts in certain tasks.^23,24,25,26^ However, studies on deep learning algorithms for analysis of cervical cytology smears have mainly analyzed only small areas of samples with instruments not suitable for POC usage. To our knowledge, no research has been conducted on the analysis of digital whole-slide images of entire Papanicolaou tests, captured in more challenging real-world clinical environments.^26,27,28,29^ Thus, this technology has not yet been applied in basic laboratories that are able to perform simple staining procedures but lack access to molecular testing, where the need for improved diagnostics is highest.^28^

In this study, we developed and implemented a novel POC digital diagnostic system at a rural clinic in Kenya, a country where cervical cancer is the leading cause of female cancer–related death.^30^ Papanicolaou smears were collected at the clinic and digitized with a portable slide scanner, and whole-slide images were uploaded to a cloud platform using the local mobile data network for development and validation of a deep learning system (DLS). We measured the diagnostic accuracy for the detection of common forms of cervical squamous cell atypia with the DLS and validated the results by comparing them with the visual assessment of samples by independent pathologists.

## Methods

Approval for the current study was issued by the Ethical Review Committee at the National Commission for Science, Technology and Innovation (Pwani University, Nacosti, Kenya). Before study participation, eligible patients were given information in English and Swahili in written and oral form about the purpose of the study and the testing procedure. Patients were allowed to ask questions and were informed that participating in the study did not in any way affect their other treatment at the clinic, and withdrawal from the study was possible at any point. Local research personnel ensured that the patients understood the information provided. After this, signed consent from patients wishing to participate was obtained. Patients were compensated for travel expenses to the sample-acquisition site and informed of the test results, but they were not offered monetary compensation for study participation. This proof-of-concept diagnostic accuracy is reported in accordance with the Standards for Reporting of Diagnostic Accuracy (STARD) reporting guideline.

### Study Design, Patient Cohort, and Collection of Samples

**Figure 1.**
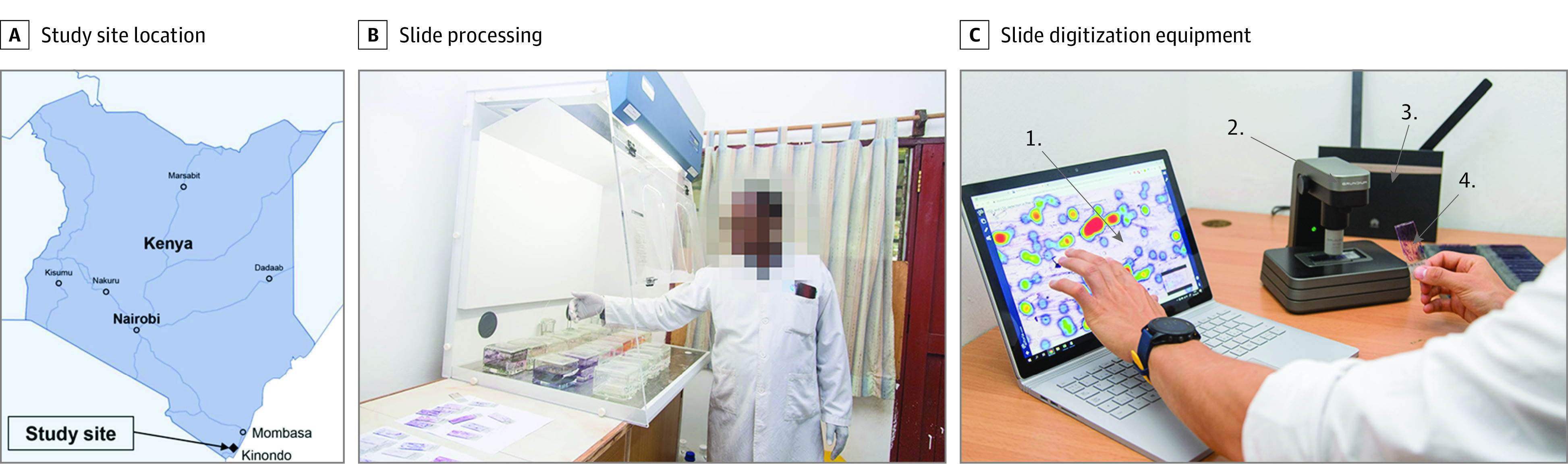
Practical Aspects of the Study Methodology A, Study site location in Kenya. B, Slide processing, including staining bench and hood. C, Slide digitization equipment, including (1) laptop computer with access to the slide-management platform, (2) slide scanner, (3) mobile-network router, and (4) Papanicolaou test microscopy slide.

The research site for this study was a local clinic (Kinondo Kwetu Health Services Clinic, Kinondo, Kwale County) in rural Kenya (approximately 40 km south of Mombasa) (Figure 1). Papanicolaou smears were acquired from 740 women attending a regional HIV-control program (eFigure in the Supplement) between September 1, 2018, and September 30, 2019, from patient volunteers who fulfilled the inclusion criteria (nonpregnant, aged between 18 and 64 years, confirmed HIV positivity, and signed informed consent acquired) (eTable 1 in the Supplement). Eligible patients were assigned a study number, after which Papanicolaou tests were obtained from the patients by trained nurses and fixed and stained with the Papanicolaou staining method (eAppendix 1 in the Supplement).^31^ After this, the staining quality was evaluated by light microscopy, after which the slides were digitized in the laboratory adjacent to the sample collection room at the research site. The slides were then stored in slide boxes and transported to the pathology laboratory (Coast Provincial General Hospital, Mombasa, Kenya). Patient records were stored digitally using the secured and password-protected web-based data-collection software REDCap (Vanderbilt University), running on a password-protected, encrypted local server in a locked room. Paper forms with patient data were stored in locked cabinets in a locked room at the clinic, accessible only to study personnel. Both digitized and physical slides were pseudonymized using study numbers, and no personal identifiers were uploaded to the cloud-based image-management platform. In cases of abnormal Papanicolaou tests, treatment expenses were covered by study funding, and treatment was arranged by a gynecologist (J.M.) in accordance with national guidelines.^32^

### Digitization of Slides at the Research Site

After the acquisition and staining of samples, Papanicolaou smears were digitized with a portable whole-slide microscope scanner (Grundium Ocus [Grundium]) (Figure 1) and deployed in a laboratory space adjacent to the room at the local clinic where the samples were collected. The device features an 18-megapixel image sensor with a 20× objective (numerical aperture: 0.40) and captures images with a pixel size of 0.48 μm. The microscope scanner was connected to a laptop computer over a wireless local area network connection and operated via the web browser interface, Chrome (Google). The coarse focus for the scanner is adjusted manually, after which the built-in autofocus routine is used for fine focus. Image files were saved on the local computer in tagged image file format and converted to the wavelet file format (Enhanced Compressed Wavelet [Hexagon Geospatial]) using a compression ratio (1:16) that was previously shown to preserve sufficient detail to not significantly alter the image-analysis results,^33^ before uploading to the image-management and machine learning platform, Aiforia Hub (Aiforia Technologies Oy). Uploading of slides was performed primarily via the 3G and 4G mobile-network router, Huawei B525S (Huawei Technologies), operating on the local mobile network, Safaricom, in Nairobi, Kenya, with a subset of slides uploaded via the in-house asymmetric digital subscriber line connection. The compressed size of the digitized slides ranged from 0.2 GB to 0.8 GB, resulting in a turnaround time for sample uploading of approximately 10 to 40 minutes over the mobile network (upload speed 0.6-1.0 MB/s) or asymmetric digital subscriber line connection (upload speed 0.6-1.2 MB/s) at the research site.

### Development of a DLS for Detection of Cervical Cell Atypia

**Figure 2.**
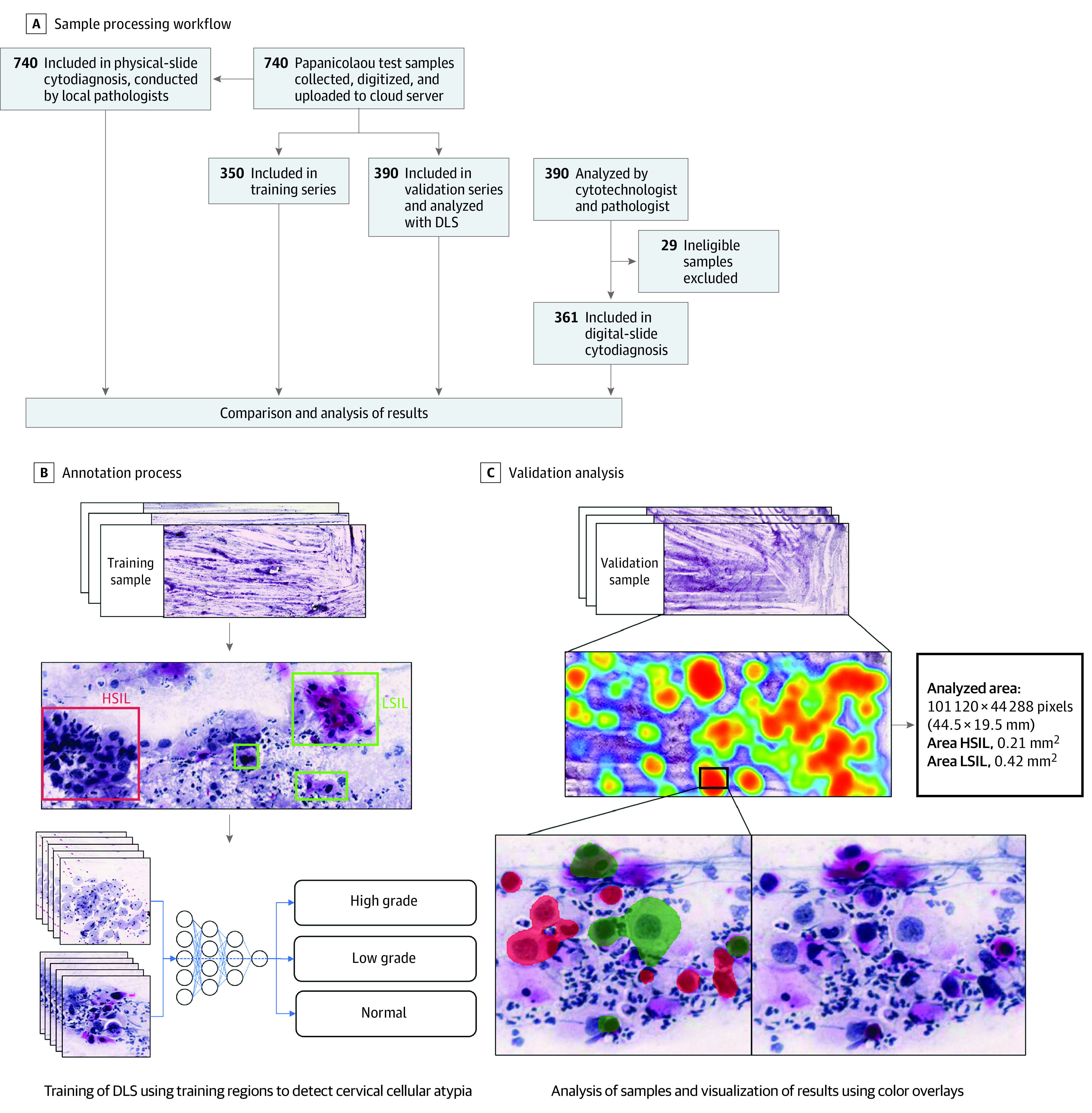
Overview of Sample Processing and Algorithm Training and Validation A, Flowchart illustrating the sample-processing workflow, showing stages from the collection of samples to the analysis of digital images and physical slides. B, Schematic view of the annotation process used for creation of the digital-slide data for training of the deep learning system (DLS). C, Validation analysis of a digitized image of a whole slide (Papanicolaou test) with the DLS, showing calculations of areas of atypia, with locations of atypia in a heatmap of the digital slide, and identification of individual cells, with color overlays (red for high-grade atypia and green for low-grade atypia). HSIL indicates high-grade squamous intraepithelial lesions; LSIL, low-grade squamous intraepithelial lesions.

To develop a DLS for the detection of cervical cell atypia in the digitized Papanicolaou smears, we used a commercially available machine learning and image-analysis platform, Aiforia Create (Aiforia Technologies). Using this platform, we trained an algorithm based on deep convolutional neural networks to detect low-grade squamous intraepithelial lesions (LSILs) and high-grade squamous intraepithelial lesions (HSILs) in the Papanicolaou smear digital whole slides. The samples series was split into a training and tuning set (n = 350) and a validation set (n = 390). Digitized slides measured approximately 100 000 × 50 000 pixels, corresponding to roughly a standard microscope glass slide (25 mm × 50 mm); ie, the entire Papanicolaou smear was scanned. Training was performed by a researcher (O.H.) assisted by a cytotechnologist specialized in cervical cytology screening, using manually defined representative regions of the digitized slides of the training series (Figure 2). Regions (n = 16 133, with cross sections of approximately 25-100 μm) were annotated visually and included areas of both normal cervical cellular morphology and various degrees of atypia. Training of the DLS used 30 000 iterations with a predetermined feature size of 30 μm, a weight decay parameter of 0.0001, 20 minibatches, a learning rate of 0.1, and 1000 iterations without progress as the early-stop limit. Training data were augmented by using image perturbations. Access to the trained model is possible remotely to analyze samples directly at the POC. Detailed configurations and hyperparameters for training of the model are described in eAppendix 1 in the Supplement.

### Expert Visual Analysis of Samples

The analysis of physical slides was performed at the pathology laboratory at Coast Provincial General Hospital (Mombasa, Kenya) with light microscopy and performed by a trained pathologist (N.M.). Slides classified as inadequate were excluded from the validation series (n = 29) (eFigure in the Supplement). Slides that were adequate for analysis (n = 361) were reviewed by the pathologist according to the Bethesda classification system.^34^ For the analyses in this study, slides with findings recorded in the cytological report as LSIL or higher (ie, HSIL or higher) were included as slides with significant cervical cell atypia. The expert assessment of the digital slides was performed by remotely located, independent experts. For this process, all digital slides in the validation series were initially screened by a cytotechnologist with experience in cervical cytology screening, and digital slides with detected cellular atypia were reviewed by a pathologist with experience in Papanicolaou test analysis (L.K.). In accordance with generally accepted quality-control guidelines for cervical cytology screening,^35^ 10% of slides that were assessed as negative in this initial cytological screening were randomly selected and submitted for re-evaluation by the pathologist. The samples were reviewed by the 2 pathologists independently without access to results from the other pathologist or the DLS.

### Statistical Analysis

General-purpose Stata statistical software, version 15.1 (StataCorp LLC) was used for analysis of the results. Statistical power calculations were performed with a sample-size formula,^36^ assuming a mean (SD) prevalence (P*_r_*) of 8% (2%) for significant atypia in the study population^37^ with an α level of .05 (and correspondingly *Z*_1−α/2 _= 1.96) and a precision parameter (ε) of 0.10:
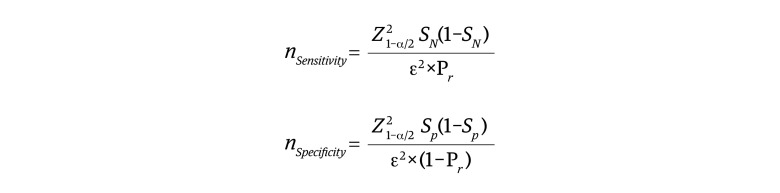
,where *S_N_* represents anticipated sensitivity; *S_P_*, anticipated specificity; and *Z*_1−α/2_, the standard normal deviate corresponding to α.

These calculations indicated a required target sample size of 304 for sensitivity and 19 for specificity with the assumed disease prevalence. All statistical tests were 2-sided unless otherwise stated, and the results were reported with 95% CIs. Evaluation of the performance of the algorithm was performed by calculating the area under the receiver operating characteristic curve (AUC) after plotting the measured true-positive rate (sensitivity) vs the false-positive rate (1 − specificity) for different thresholds of slide-level positivity. Interobserver agreement was measured using κ statistics.

## Results

### Detection of Cervical Cell Atypia in Digital Papanicolaou Smears With the DLS

**Figure 3.**
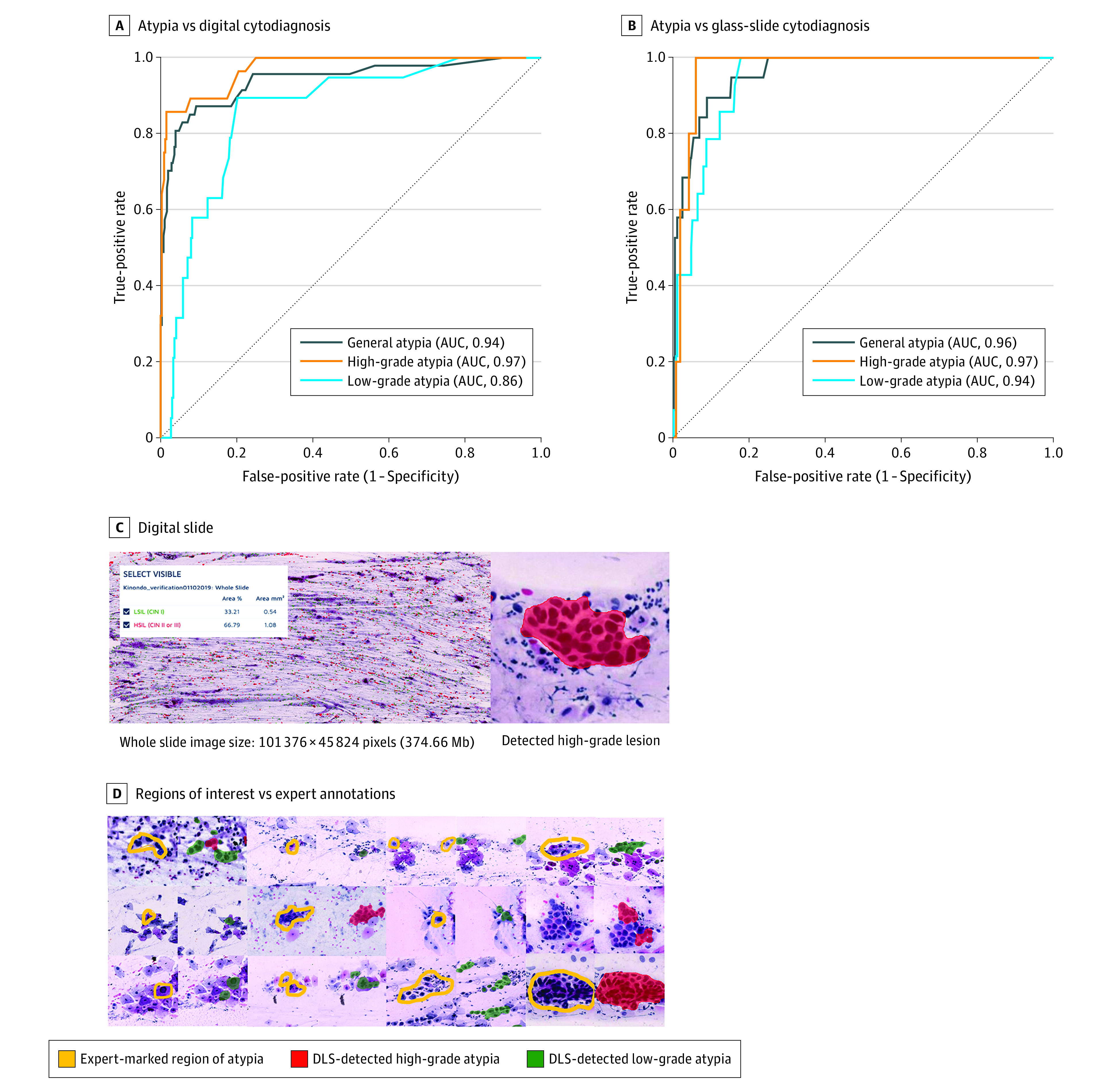
Detection of Atypia in Cervical Smears by Automated Deep Learning System (DLS) and by Manual Assessment Areas under the receiver operating characteristic curves (AUCs) for the detection of general atypia, high-grade atypia, and low-grade atypia with the DLS compared with manual assessment of digital slides by a cytotechnologist and a pathologist (A) and physical slides by a local pathologist (B). Receiver operating characteristics curves were calculated for a range of operating thresholds for the DLS. C, View of a digitized sample on the cloud-based slide-management platform, with a magnified view of a detected atypical cellular cluster at 40× digital magnification. D, Examples of atypical cells marked by the experts in the digitized slides (yellow) and the corresponding regions extracted from the DLS results, with cells assessed as high-grade atypia colored in red and low-grade atypia colored in green.

Papanicolaou tests from 740 HIV-positive women (mean [SD] age, 41.8 [10.3] years) were collected. Following the training of the DLS and exclusion of 29 inadequate slides (7%) classified as unevaluable by the local pathologist (eAppendix 1 in the Supplement), 361 slides remained in the validation series (average size, 100 387 × 47 560 pixels). The expert assessment of digitized slides revealed 19 slides (5%) with low-grade atypia, 28 slides (8%) with high-grade atypia, and 314 slides (87%) that were negative for significant squamous cell atypia (defined as atypical squamous cells of undetermined significance or lower). With these results as reference, the DLS achieved a classification accuracy for general atypia as measured by AUC of 0.94, a sensitivity of 95.7% (95% CI, 85.5%-99.5%), and a specificity of 84.7% (95% CI, 80.2%-88.5%) at the selected threshold (Figure 3). The AUC for detection of slides containing HSILs or higher-grade lesions was 0.97, with a sensitivity of 85.7% (95% CI, 67.3%-96.0%) and a specificity of 98.5% (95% CI, 96.5%-99.5%). For the detection of slides containing only LSILs, the AUC was 0.86, with a sensitivity of 84.2% (95% CI, 60.4%-96.6%) and a specificity of 86.0% (95% CI, 81.8%-89.5%) (Table). In these analyses, slides with discrepancies in the type of atypia (such as low-grade slides that were classified as high grade, or vice versa) were considered to be of equal statistical value to slides with atypia that were classified as negative. Overall, the DLS classified 266 slides (74%) as negative, 61 slides (17%) as positive for low-grade atypia, and 34 slides (9%) as positive for high-grade atypia. Compared with the expert assessment of the digital slides, 2 slides with low-grade atypia, but no high-grade slides, were falsely classified as negative by the DLS (<1%). Four slides (1%) with high-grade atypia were classified as low grade by the DLS (Table). The negative predictive value was high for general atypia (266 of 268 [99.3%; 95% CI, 97.3%-99.9%]), low-grade atypia (294 of 297 [99.0%; 95% CI, 97.1%-99.8%]), and high-grade atypia (328 of 332 [98.8%; 95% CI, 96.9%-99.7%]). The measured interrater agreement between the DLS and visual scoring of the digital slides was substantial (κ = 0.72; 95% CI, 0.62-0.82; *P* < .01) (eTable 2 in the Supplement).

**Table.** Detection of Cervical Cell Atypia With the Deep Learning System in Digitized Papanicolaou Tests, Compared With Expert Assessments of Digitized and Physical Slides^a^

Diagnostic comparison	% (95% CI)		No. (%)			
			True		False	
	Sensitivity	Specificity	Positive	Negative	Positive	Negative
**Digitized-slide cytodiagnosis**						
General atypia	95.7 (85.5-99.5)	84.7 (80.2-88.5)	45 (12.5)	266 (73.7)	48 (13.3)	2 (0.6)
High-grade atypia	85.7 (67.3-96.0)	98.5 (96.5-99.5)	24 (6.6)	328 (90.9)	5 (1.4)	4 (1.1)^b^
Low-grade atypia	84.2 (60.4-96.6)	86.0 (81.8-89.5)	16 (4.4)	294 (81.4)	48 (13.3)	3 (0.8)
**Glass-slide cytodiagnosis**						
General atypia	100.0 (82.4-100.0)	78.4 (73.6-82.6)	19 (5.3)	268 (74.2)	74 (20.5)	0
High-grade atypia	100.0 (47.8-100.0)	93.3 (90.1-95.6)	5 (1.4)	332 (92.0)	24 (6.6)	0
Low-grade atypia	21.4 (4.7-50.8)	82.4 (78.0-86.3)	3 (0.8)	286 (79.2)	61 (16.9)	11 (3.0)^c^

^a^ Sensitivity and specificity results from the deep learning system are shown with the associated 95% CIs. Numbers of false-negative, false-positive, true-negative, and true-positive assessments are shown with the corresponding percentage of the total number of slides in the validation series (n = 361).

^b^ Four slides identified as having high-grade atypia were classified as low-grade atypia by the deep learning system.

^c^ Eleven slides identified by the local pathologist as low-grade atypia were classified as high-grade atypia by the deep learning system.

### Comparison of DLS Results With the Pathologist Glass-Slide Cytodiagnosis

Next, we evaluated the performance of the DLS as compared with the assessment of physical slides by the local pathologist. The report from the pathology laboratory classified 342 slides (95%) as negative for significant squamous cell atypia, 14 (4%) as positive for low-grade atypia, and 5 (1%) as positive for high-grade atypia. With reference to these results, the DLS achieved high sensitivity for general atypia (100%; 95% CI, 82.4%-100%) and for high-grade atypia (100%; 95% CI, 47.8%-100%), with corresponding specificities of 78.4% (95% CI, 73.6%-82.4%) and 93.3% (95% CI, 90.1%-95.6%), respectively (Table). Specificity was moderate for low-grade atypia (82.4%; 95% CI, 78.0%-86.3%), but sensitivity was lower (21.4%; 95% CI, 4.7%-50.8%) because 11 of the 14 slides that were classified as low-grade atypia in the cytological report from the pathology laboratory were classified as high-grade atypia by the DLS. The interrater agreement between the DLS and the physical slide assessment was fair (κ = 0.36; 95% CI, 0.24-0.49; *P* < .01) (eTable 2 in the Supplement), but no atypical slides were falsely classified as negative by the DLS. The DLS achieved high AUCs for detection of general atypia (0.96), high-grade atypia (0.97), and low-grade atypia (0.94) (Figure 3). The negative predictive value was high for general atypia (266 of 266 [100%; 95% CI, 98.6%-100.0%]), high-grade atypia (332 of 332 [100%; 95% CI, 98.9%-100.0%]), and low-grade atypia (286 of 297 [96.3%; 95% CI, 93.5%-98.1%]) (Table).

## Discussion

In this study, we implemented a POC digital diagnostics system at a peripheral clinic in Kenya and evaluated it for the analysis of Papanicolaou smears. The DLS achieved high accuracy for the detection of cervical squamous cell atypia, with AUCs of 0.94 to 0.96 and sensitivities of 96% to 100%, compared with the visual interpretation of digitized and physical slides. With the visual assessment of digitized slides as a reference, the number of false-negative assessments by the DLS was low, with 2 low-grade slides incorrectly classified as negative (although 4 high-grade slides were falsely classified as low grade). Compared with the visual analysis of the physical slides by the local pathologist, the DLS sensitivity was high for general atypia (100%) and high-grade atypia (100%) but low for low-grade atypia (21%), given that 11 of 14 physical slides that were assessed as low grade were classified as high grade by the DLS. The visual interpretation of Papanicolaou smears is known to be subjective, especially when assessing low-grade findings,^10,38^ and accordingly, we observed variation between the experts’ assessments of slides, with a lower threshold for the classification of findings as high grade by the pathologist who assessed the digitized slides. The DLS was trained with assistance from the experts who analyzed the digital slides, which possibly explains why the DLS classification showed higher agreement compared with these results. Notably, however, none of the slides that were classified as negative by the DLS were classified as atypical in the cytodiagnosis of the physical slides. Previous studies have reported encouraging results with the deep learning–based analysis of smaller cropped images from Papanicolaou smears^26,27,29,39^ that were digitized with conventional slide scanners, but clinical application requires the examination of substantially larger sample areas.^28^ In this study, we used routine samples collected at the clinic, and correspondingly, the whole-slide images were magnitudes larger than those previously analyzed, measuring on average 100 387 × 47 560 pixels; thus, the total number of pixels analyzed corresponded to approximately twice the number in the entire ImageNet database (>14 million images of everyday objects) at commonly used resolutions.^40^ Papanicolaou smears may contain very limited numbers of isolated atypical cells, and robust algorithms are necessary to reliably detect such cells in these large and complex samples. In this study, we investigated the use of a DLS as a potential screening tool with a relatively low threshold for the classification of slides as atypical, to ensure high sensitivity at the potential expense of specificity; this method resulted in relatively high rates of false-positives for low-grade atypical slides. However, because this type of algorithm can operate using multiple configurations, sensitivity and specificity could be adjusted to match clinical requirements, with high sensitivity for screening purposes or higher specificity for confirmatory diagnostics. Importantly, our findings demonstrate how a frontline diagnostic system based on POC digital microscopy with deep learning–based analysis of microscopy slides can be deployed in rural clinical settings. As the DLS can be accessed remotely, the proposed system enables an end-to-end pipeline for digital analysis of samples at the POC. To our knowledge, no other study has evaluated this technology using whole slides that have been collected, stained, digitized, and uploaded using a mobile data network in similar settings. Overall, we achieved high negative predictive values for the detection of atypical slides in these demanding settings, suggesting that the method may be useful for screening purposes in resource-limited environments. For this application, clinical implementation could reduce sample analysis workloads to allow clinicians to focus on verifying potentially abnormal slides and could exclude most slides (approximately 70%) while retaining high sensitivity for atypical slides. By combining this technology with primary POC molecular testing for HPV,^8,14^ the number of slides that needs to be analyzed could be reduced even further, which would be essential in low-resource areas where the number of practicing pathologists is low and the cervical cancer incidence is increasing.^4,41^ By using methods such as self-sampling for both molecular- and cytology-based testing,^42^ the dissemination of tests to large populations could be feasible. Although the final cost of implementing a system like this is not yet possible to determine precisely, we estimate the per-sample equipment and reagent costs to be in the range of $2.00 to $5.00 US dollars (eAppendix 2 in the Supplement). As this technology provides a platform for general-purpose digital microscopy, it is likely to be applicable also for diagnostics of other diseases that are common in resource-limited areas and high-risk populations, such as neglected tropical parasites,^43^ sexually transmitted infections,^44^ and malignant neoplasms.^45,46,47^ In this way, opportunities are created for integrated disease control.

### Limitations

Because this is an early study, it has limitations. The DLS was benchmarked against 2 independent experts for the assessment of samples, but for the results to be directly comparable with other screening modalities, the ideal reference standard would be cervical biopsies with histologically confirmed precancers, which were not available here. Owing to the subjective nature of Papanicolaou smear cytology, this means that the results from both experts are not directly comparable with each other. Furthermore, even though the total number of slides collected was relatively large, the prevalence of slides with significant atypia was limited. Although these results are promising, increasing the amount of training data would likely improve the performance of the DLS and would be required before confirmatory diagnostic applications. Moreover, as this was a single-center study, the results might differ if the sample acquisition and preparation procedures are altered, and further work is needed to prospectively validate these results. Furthermore, because we evaluated only Papanicolaou smears from HIV-positive women, the results might differ owing to varying levels of prevalence in other populations (eAppendix 2 and eTable 3 in the Supplement).

## Conclusions

In this diagnostic study, we developed a new system for deep learning–based digital microscopy at the POC, which was used for the analysis of cervical smears in cervical cancer screening. Results suggest that the detection of squamous cell atypia with the technology was feasible, with high sensitivity for slides demonstrating atypia, particularly for slides showing high-grade atypia. The clinical utilization of this technology could reduce the sample analysis workload for microscopists and provide a platform for general-purpose digital pathology, which is implementable in rural areas. As such, the technology here could create new opportunities to facilitate the diagnostics of a variety of diseases that are still underdiagnosed, especially in low-resource settings.

## Notes

Supplement. **eAppendix 1.** Additional Details About Study Methodology, Including Preparation of Samples and Training of the Deep-Learning Model **eAppendix 2.** Supplementary Information **eFigure.** CONSORT-Style Flowchart of Processing of Samples in the Study **eTable 1.** Epidemiological Information and Characteristics of Patient Cohorts **eTable 2.** Cross-Tabulation of Interobserver Agreement Between Observers With Kappa Statistics **eTable 3.** Extrapolated Negative and Positive Predictive Values Depending on Disease Prevalence
