# Supplementary material for: Point-of-Care Digital Cytology With Artificial Intelligence for Cervical Cancer Screening in a Resource-Limited Setting
Source: JAMA Netw Open. 2021 Mar 17;4(3):e211740. doi: 10.1001/jamanetworkopen.2021.1740 (PMC7970338; doi:10.1001/jamanetworkopen.2021.1740)
Supplement: Supplement. — eAppendix 1. Additional Details About Study Methodology, Including Preparation of Samples and Training of the Deep-Learning Model eAppendix 2. Supplementary Information eFigure. CONSORT-Style Flowchart of Processing of Samples in the Study eTable 1. Epidemiological Information and Characteristics of Patient Cohorts eTable 2. Cross-Tabulation of Interobserver Agreement Between Observers With Kappa Statistics eTable 3. Extrapolated Negative and Positive Predictive Values Depending on Disease Prevalence [file jamanetwopen-e211740-s001.pdf]

Supplementary Online Content

Holmström O, Linder N, Kaingu H, et al. Point-of-care digital cytology with artificial intelligence for cervical cancer screening in resource-limited setting. *JAMA Netw Open*. 2021;4(3):e211740. doi:10.1001/jamanetworkopen.2021.1740

eAppendix 1. Additional Details About Study Methodology, Including Preparation of Samples and Training of the Deep-Learning Model
eAppendix 2. Supplementary Information
eFigure. CONSORT-Style Flowchart of Processing of Samples in the Study
eTable 1. Epidemiological Information and Characteristics of Patient Cohorts
eTable 2. Cross-Tabulation of Interobserver Agreement Between Observers With Kappa Statistics
eTable 3. Extrapolated Negative and Positive Predictive Values Depending on Disease Prevalence

This supplementary material has been provided by the authors to give readers additional information about their work.

eAppendix 1. Additional Details About Study Methodology, Including Preparation of Samples and Training of the Deep-Learning Model

Recruitment.
1. Patients who met the Project's inclusion criteria were recruited as outlined in the study protocol and assigned a Study Number
2. A Gynecological sample Collection Form and Pathologist Report Forms were prepared for each patient. The forms were filled with the Patient's personal identification data, Unique Number and CCC Number.
3. A Pap sample was collected from the patient, applied on a clean frosted scratch-free glass slide and labeled with the Patient's Unique Number and the CCC Number. It was immediately flooded with the sachet fixative provided in the Pap kit and allowed to dry in air.

Eligible patients were assigned a study number, after which Pap smears were obtained from the patients using a cervical broom sampling kit (Touchfree Cytopak, AS Diagnostics & Disposables, Chennai, India) by trained nurses. The cervical sample was applied to a clean frosted glass slide, fixated using the provided fixative solution, air-dried at room temperature and fixed in 95% ethyl alcohol for 15 minutes at room temperature after which staining was performed with the Papanicolaou staining method described below.

Staining Preliminaries.
The Pap staining procedure was carried out in a fumes hood using glassware, reagents and the appropriate accessories of high quality. Approved methodology and good laboratory practices were maintained throughout the project.

Staining
The slides were arranged in staining racks and further fixed in 95% ethyl alcohol for 15 minutes, then dipped in 70% and 50 % ethyl alcohol solutions for 5 minutes in each. The slides were rinsed in distilled water for 3 minutes. Staining was done in Meyers haematoxylin stain for 7 minutes. The smears were washed in distilled water for 7 minutes. They were dipped in two changes of 95% ethyl alcohol 3 minutes each. First counterstain was done in orange gold (OG-6) stain for 5 minutes and thereafter slides washed in two changes of 95% ethyl alcohol for 3 minutes each. Second counterstain was carried out in eosin azure (EA-65) stain for 5 minutes.
Final wash was done in two changes of 95% ethyl alcohol 3 min each. The smears were dehydrated in two changes of absolute ethanol 2 minutes each.
Clearance was carried out in two baths of rectified Xylene 10 minutes each. The smears were covered by mounted coverglass on DPX and dried at room temperature overnight. The slides were examined under the microscope to assess process quality, scanned and uploaded.

Pathologist
Each scanned slide's label was counter-checked against the Study Unique Number and the CCC Number for the patient as recorded in the Pathologists Report Form. Matched slides and forms were packaged and sent to the pathologist at The Coast General Hospital for examination.

Reports
The reports were collected from the pathologist and entered in Redcap computer program and recorded in the project register.
Reports summary
The Pathologist categorised samples as suitable or unsuitable for evaluation.
A smear was unsuitable for evaluation if it:-
i) had insufficient cellular material
ii) was glossily blood stained
iii) had purulent discharge, excess mucus or inflammatory cells covering >90% of details
iv) had no endocervical cells
v) had sub-optimal processing.

Overall, 8% of samples were excluded due to inadequate quality for assessment, due to the reasons previously stated. Overall, the prevalence of inflammatory samples was relatively high (approximately 40%); and furthermore sample-technical problems with the preparation of samples might have contributed in the beginning of the study, as fewer inadequate samples were observed towards the end of the study.

Samples which were suitable were evaluated and results presented according to the Bethesda classification system:
i) Negative for intraepithelial lesions or malignancy
ii) inflammatory
iii) B. vaginosis
iv) Cellular atypia
v) Cytodiagnosis where applicable.
vi) Atrophic

© 2021 Holmström O et al. *JAMA Network Open.*

Training of the deep-learning system (DLS)

We used a commercially available machine-learning and image-analysis platform (Aiforia Create, Aiforia Technologies, Helsinki, Finland). The software is commercially available at: https://www.aiforia.com/aiforia-create/. The samples were split with a 50–50 distribution of the target number of samples into the training series (n = 350), used for training and tuning of the model, and external validation series (n = 390), used for validation of the algorithm. On the slide-management platform, digitized slides are stored as JPG-compressed tile-maps with a pyramid-structure of zoom levels (70% JPG-quality). Access to the image server for remote slide viewing was established with a web browser secured with Secure Socket Layer encryption. Individual digitized slides measured approximately 100,000 × 50,000 pixels. Training was performed by a researcher (OH), assisted by a cytotechnologist (KK) specialized in cervical-cytology screening, using manually defined representative regions (n = 16,133; cross-sections of ~25–100 µm) of the digitized slides of the training series. Regions were selected visually and included areas of both normal cervical cellular morphology and various degrees of atypia. Training of the DLS used 30,000 iterations with a pre-determined feature size (field-of-view) of 30 µm. We used a weight decay parameter of 0.0001, 20 mini-batches per iteration (mini-batch size: 40), a learning rate of 0.1 and 1000 iterations without progress as the stop limit for the training. To increase generalizability of the algorithm, the training data were augmented with the following image perturbations: variation in scale (± 10%), aspect ratio (± 10%), shear distortion (± 10%), luminance (± 10%), contrast (± 10%), white balance (± 10%) and variation in image compression quality (40–60%). Selection of hyperparameters were performed based on the results during the training, i.e. based on performance of the model for analysis of samples in the training series. DLS analyses were performed on entire digitized slides, and a slide-level operating threshold for the total area of detected atypia was decided on the basis of the training data, to determine whether slides were classified as atypical, decided on based on best performance in the training data set. Analysis time for one whole-slide image with the trained model was ~30 s. The image analysis gave the total area per slide of LSILs and HSILs (or higher-grade lesions). For the slide-level classification by the DLS, slides with both detected low- and high-grade lesions where classified as high-grade.

© 2021 Holmström O et al. *JAMA Network Open.*

eAppendix 2. Supplementary Information

Estimation of price per sample for the proposed system

For this study, we acquired the materials for the preparation of samples in bulk from local vendors. The digital equipment required (laptop computer, slide scanner, mobile network router and 4G data subscription) were acquired from Finland and transported to the research site, except for the 4G data subscription (Safaricom, Kenya). By assuming that the system can be used to analyse 10,000 samples, we calculated the per-sample equipment and reagent costs to approximately 4.60 USD. This includes the price of the equipment to prepare slides (glass slide, cervical broom sampling kit and fixative solution; 1.73 USD), staining reagents (1.87 USD) and the price of the equipment for the digital laboratory. If we reasonably assume that the system would allow for digitization of 100,000 samples, the per-sample equipment and reagent costs would be reduced to approximately 3.70 USD. Wider-scale analysis would likely also allow the reduction of the prices for sample-preparation materials, and based on these assumptions, it seems reasonable to assume that a per-sample equipment and reagent costs of 2-5 USD would be achievable. In comparison, the price charged per patient for a Pap smear test currently in Kwale county is approximately 2000-3000 KES (18 – 27 USD), with the pathologist charging an additional fee per sample (here, approximately 700 KES, or 6.30 USD, per sample).

Extrapolated results for populations with different prevalences of cervical atypia

In our study we achieved a prevalence of approximately 7.65% (4.8-10.5%) of atypical Pap smears. As we studies samples only from HIV-positive women, it is likely that the prevalence observed here differs from the general population, as HIV-infection is associated with an increased risk of cervical cellular atypia and a higher prevalence of abnormal smears (1-4). Overall, the prevalence of cervical epithelial atypia varies significantly between populations. According to the literature, the prevalence of atypia (LSIL or higher) varies from approximately 3% to even 25% in certain populations (5). Although the reported prevalence among HIV-positive women is significantly higher, cervical epithelial atypia is relatively common (11%) also among HIV-negative women e.g. in e.g. Nigeria (11%) (6). Compared to general populations, the observed prevalence in our study population was higher (8% compared to approximately 5%) (5). Using the measured levels of sensitivity and specificity in this study, we have extrapolated the results in terms of positive and negative predictive values for populations with varying levels of cervical epithelial atypia. Notably, even though the positive predictive value is decreased with lower disease prevalences, the negative predictive value remains high (> 99%) even for populations with different levels of prevalence (4-24%).

(1) Muhammad Z, Usman I, Datti Z, Avidime A, Danjuma S, Taoheed A, et al. Incidence and risk factors of cervical dysplasia among human immune deficiency virus positive and human immune deficiency virus negative women at Aminu Kano Teaching Hospital. Sahel Med J (4):160.

(2) Getinet M, Gelaw B, Sisay A, Mahmoud EA, Assefa A. Prevalence and predictors of Pap smear cervical epithelial cell abnormality among HIV-positive and negative women attending gynecological examination in cervical cancer screening center at Debre Markos referral hospital, East Gojjam, Northwest Ethiopia. BMC Clinical Pathology 2015;15(1):16.

(3) Teixeira NCP, Araújo A,Cristina Labanca, Correa CM, Lodi, Claudia Teixeira da Costa, Lima MIM, Carvalho NdO, et al. Prevalence and risk factors for cervical intraepithelial neoplasia among HIV-infected women. Brazilian Journal of Infectious Diseases 2012;16:164-169.

© 2021 Holmström O et al. *JAMA Network Open.*

(4) Memiah P, Mbuthia W, Kiiru G, Agbor S, Odhiambo F, Ojoo S, et al. Prevalence and Risk Factors Associated with Precancerous Cervical Cancer Lesions among HIV-Infected Women in Resource-Limited Settings. AIDS Research and Treatment 2012;2012:953743.

(5) Ting J, Rositch AF, Taylor SM, Rahangdale L, Soeters HM, Sun X, et al. Worldwide incidence of cervical lesions: a systematic review. Epidemiol Infect 2015;143(2):225-241.

(6) Akinfolarin AC, Olusegun AK, Omoladun O, Omoniyi-Esan G, Onwundiegu U. Age and Pattern of Pap Smear Abnormalities: Implications for Cervical Cancer Control in a Developing Country. Journal of cytology 2017 Oct;34(4):208-211.

© 2021 Holmström O et al. *JAMA Network Open.*

eFigure. CONSORT-Style Flowchart of Processing of Samples in the Study

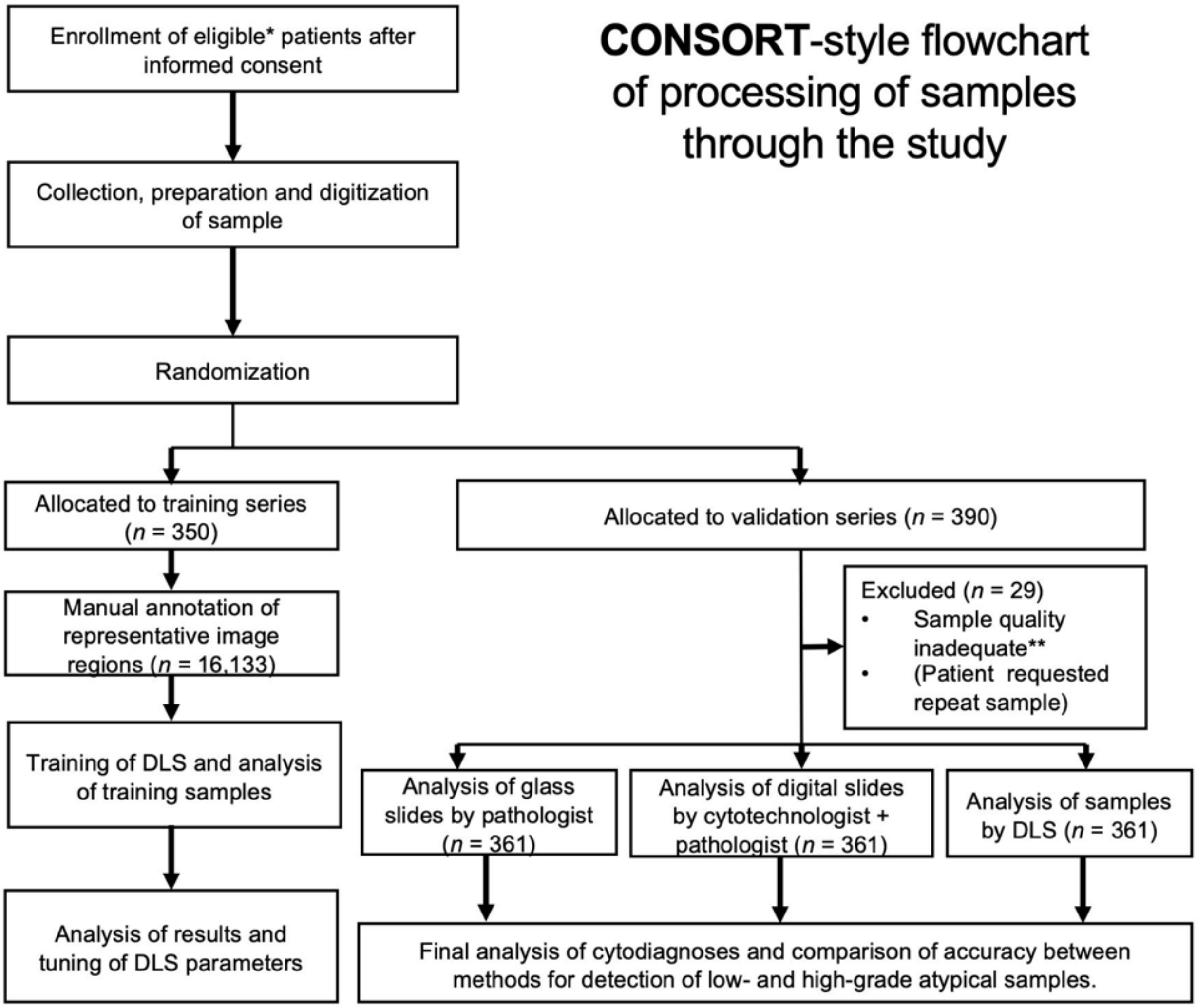

*Study inclusion criteria were: non-pregnant women, age between 18 and 64, confirmed HIV-positivity, signed informed consent and no contraindications for the screening test.
**Samples excluded as unsatisfactory for evaluation when sample has insufficient cellular material, significant amount of blood, purulent discharge, mucus or inflammatory cells or technical problems with staining or preparation which prevented evaluation of sample.

Further details about the study protocol available in the attached Supplementary material.

© 2021 Holmström O et al. *JAMA Network Open.*

eTable 1. Epidemiological Information and Characteristics of Patient Cohorts

	Training	Validation	Total
Number of samples	350	361	711
Mean age, years (SD)	42.9 (14.1)	40.7 (10.0)	41.8 (12.3)
Number of children, n (%)	3.6 (2.3)	3.1 (2.0)	3.4 (2.2)
Currently smoking, n (%)	6 (1.7)	7 (1.9)	13 (1.8)
Postmenopausal, *n* (%)	109 (31.1)	95 (26.3)	204 (28.7)
Not using contraceptives, n (%)	178 (50.9)	183 (50.7)	361 (50.8)
Currently using contraceptives, n (%)	156 (44.6)	174 (48.2)	330 (46.4)
Currently receiving HRT, n (%)	0 (0)	0 (0)	0 (0)
Previously taken PAP test, n (%)	6 (1.7)	10 (2.8)	16 (2.3)
Age at first intercourse, years	17.6	18.0	17.8
Years since HIV diagnosis (mean; calculated since September 2018)	5.29	5.08	5.17
WHO stage 3 or higher at diagnosis, *n* (%)	36 (10.3)	37 (10.2)	73 (10.3)
Initial CD4 count at diagnosis, cells/µL (SD)	404 (284)	403 (294)	404 (289)
Initial VL at HIV diagnosis, copies/mL (SD)	18,000 (110,000)	2,700 (140,000)	9,000 (70,000)
High VL control (>1000 copies/mL) measured at last recent control, *n* (%)	36 (10.3)	19 (5.3)	55 (7.7)
Low VL measured at last control (<1000 copies/mL), *n* (%)	231 (66.0)	267 (74.0)	498 (70.0)
Unstable HIV balance*, n (%)	70 (20.0)	100 (27.7)	170 (23.9)
Digital slide cytodiagnosis, n (%):			
No significant atypia (%)	323 (92.3)	314 (87.0)	637 (89.6)
Low-grade atypia (LSIL) (%)	13 (3.7)	19 (5.3)	32 (4.5)
High-grade atypia (HSIL, or higher) (%)	15 (4.3)	28 (7.8)	43 (6.0)
Glass slide cytodiagnosis, n (%):			
No significant atypia (%)	337 (96.3)	342 (94.7)	679 (95.5)
Low-grade atypia (LSIL) (%)	11 (3.1)	14 (3.9)	25 (3.5)
High-grade atypia (HSIL, or higher) (%)	4 (1.1)	5 (1.4)	9 (1.3)
Signs of inflammation in sample, n (%)	164 (46.9)	151 (41.8)	315 (44.3)

Numbers of samples (slides) shown with associated percentage of total number of slides in training, validation or total sample series. When applicable, mean values with associated standard deviations calculated for patients with corresponding clinical information available.

SD = Standard deviation
HRT = Hormone replacement therapy
VL = Viral load
*Classified in patient records as 'Unstable Patient'

© 2021 Holmström O et al. *JAMA Network Open.*

eTable 2. Cross-Tabulation of Interobserver Agreement Between Observers With Kappa Statistics

**Comparison**	Deep-learning system (κ, CI95%)	Digital slide cytodiagnosis (κ, CI95%)
Deep-learning system		0.72 (0.62 - 0.82)
Glass-slide cytodiagnosis	0.36 (0.24 - 0.49)	0.50 (0.35 - 0.65)

© 2021 Holmström O et al. *JAMA Network Open.*

eTable 3. Extrapolated Negative and Positive Predictive Values Depending on Disease Prevalence

**Prevalence of atypical smears**	4%	8%*	12%	16%	20%	24%
PPV	0.290	0.461	0.573	0.652	0.710	0.7564
NPV	0.999	0.998	0.997	0.996	0.994	0.9925

PPV = Positive predictive value

NPV = Negative predictive value

© 2021 Holmström O et al. *JAMA Network Open.*
